# Synthesis and characterization of TiO_2_-V_2_O_5_-MCM-41 for catalyzing transesterification of dimethyl carbonate with phenol

**DOI:** 10.1186/s13065-018-0474-6

**Published:** 2018-10-20

**Authors:** Jinfeng Zhang, Yan Gao, Jiyao Zhang, Jianshe Zhao, Hanxi Shen

**Affiliations:** 1 Key Laboratory of Synthetic and Natural Functional Molecule Chemistry of Ministry of Education, Shaanxi Key Laboratory of Physico-Inorganic Chemistry, College of Chemistry & Materials Science, Northwest University, Xi’an, 710069 Shaanxi China; 2 Shaanxi Key Laboratory of Petroleum for Fine Chemicals, Shaanxi Provincial Research and Design Institute of Petroleum and Chemical Industry, Xi’an, 710054 Shaanxi China

**Keywords:** Diphenyl carbonate, Dimethyl carbonate, Phenol, Transesterification, TiO_2_-V_2_O_5_-MCM-41

## Abstract

A series of TiO_2_-V_2_O_5_-MCM-41 molecular sieve catalysts were prepared by the impregnation method. The prepared catalysts were characterized by different techniques including X-ray diffraction, Fourier transform infrared spectroscopy, X-ray photoelectron spectroscopy, and N_2_ adsorption–desorption. These catalysts were applied in the catalytic synthesis of diphenyl carbonate (DPC) by the transesterification of dimethyl carbonate (DMC) with phenol. The synthesis results indicated that the catalysts possessed the high specific surface area and large pore volume and included titanium with four ligands. Due to the vanadium introduction into Ti-MCM-41, the catalytic activity was promoted, by-products were reduced, and the catalytic activity and stability of the catalyst were significantly improved. With 10%V-20%Ti-MCM-41 catalyst, the optimal synthesis results including the conversion rate of DMC of 33.88%, the selectivity of DPC of 35.84%, and the yield of DPC of 12.14% were obtained.

## Introduction

Diphenyl carbonate (DPC) is a green engineering thermoplastic intermediate widely used in the formation of various organic and polymeric materials, particularly in the synthesis of polycarbonate by the melt transesterification process [1]. The synthesis processes of DPC include the phosgene processes, carbonyl-action of phenol and CO_2_, and oxidative carbonylation of phenol and transesterification [2–4].

**Scheme 1:**
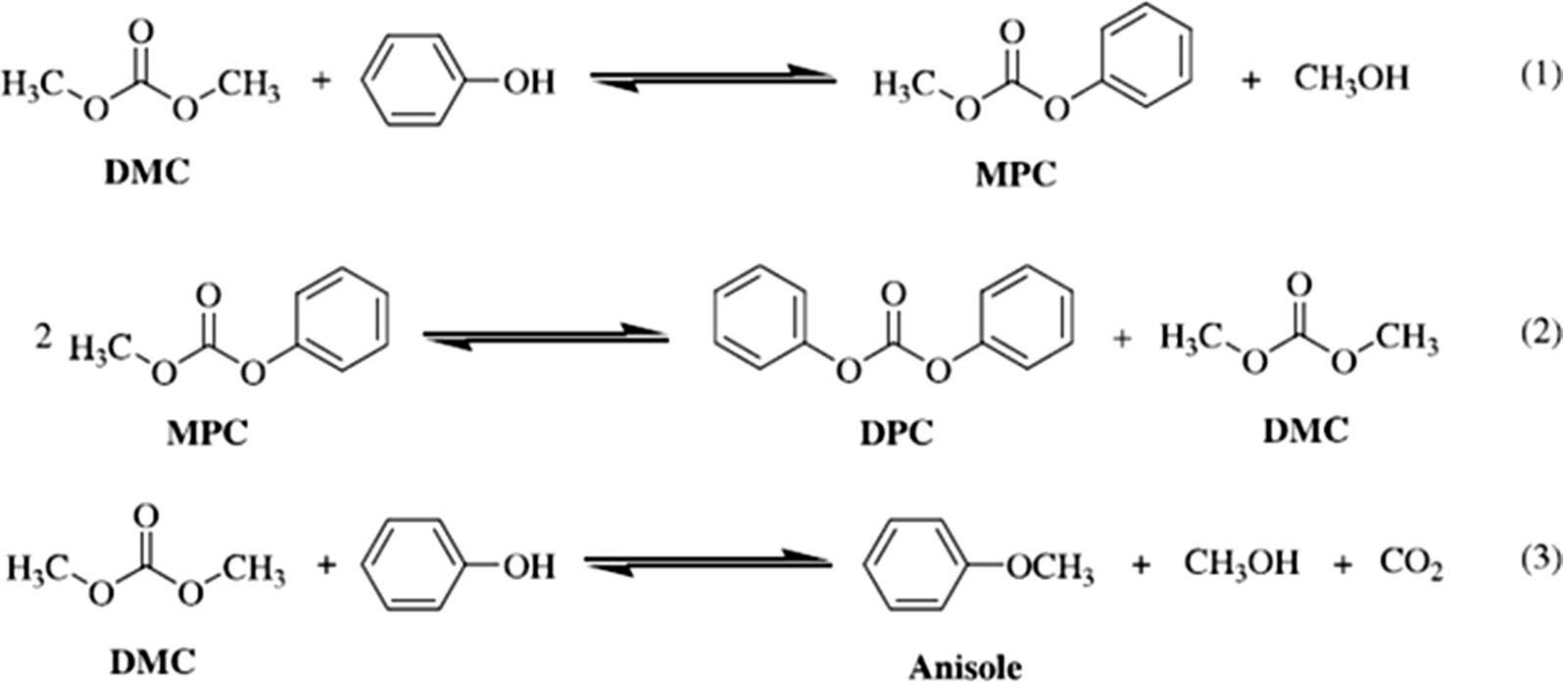
Main reactions involved from DMC to DPC

Transesterification of phenol with DMC is the most suitable method for an industrial production of DPC, which can be promoted by effective catalysts. As shown in Scheme 1, this route is a two-step process, including the transesterification of DMC and phenol to methyl phenyl carbonate (MPC) and the transesterification of MPC and phenol to DPC or the disproportion of MPC to DPC and DMC.

Recently, the heterogeneous catalytic has become a hot topic for the transesterification of DMC and phenol. The reported heterogeneous catalysts include single or composite oxides [5], zeolites [6], hydrotalcite-like compounds [7], and heteropoly compounds [8]. Tong et al. found that V_2_O_5_ had the excellent activity for the transesterification of DMC with phenol [9]. Other groups presented that Pb_3_O_4_/ZnO, PbO/MgO, and V_2_O_5_ as catalysts contributed to the synthesis of DPC [10]. Tang and coworkers proposed that TiO_2_@SiO_2_ possessed the favorable catalytic performance [11]. Zhang and coworkers reported that MoO_3_/SiO_2_ showed a high activity for both transesterification and disproportionation [12]. In 1992, the Mobil Company in the United States of America invented M41S series of mesoporous molecular sieves which showed the attractive prospect for the shape-selective catalytic oxidation of organic macromolecular materials [13]. Li reported that MoO_3_/SiMCM-41 shows prominent activity for transesterification [14].

In this study, a series of TiO_2_-V_2_O_5_-MCM-41 was designed and prepared. All the compositions were characterized by X-ray powder diffraction, Fourier transform infrared spectroscopy, X-ray photoelectron spectroscopy, and N_2_ adsorption–desorption. We further explored the catalytic performance of the transesterification reaction of DMC and phenol and verified that TiO_2_-V_2_O_5_-MCM-41 was an efficient catalyst for the transesterification of DMC with phenol.

## Experimental

### Chemical reagents

Tetraethyl orthosilicate (TEOS), cetyl trimethyl ammonium bromide (CTAB), ammonium metavanadate, tetrabutyl titanate, ethanol, ammonia water, phenol, and dimethyl carbonate were of analytical grades and directly used.

### Preparation of catalysts

#### Preparation of Si-MCM-41

The MCM-41 was synthesized based on the previous method [15]. Firstly, 2.0 g CTAB was dissolved in 65.0 g deionized water at a constant temperature of 40 °C. After stirring the mixture for half an hour, 20.0 g ammonia water was added into the solution, followed by 30-min stirring. Then, 8.5 g TEOS was dropped slowly into the mixture for 2-h stirring. After the reaction, the resulting product was aged at ambient temperature for 24 h and then crystallized at 110 °C for 24 h in the reaction kettle. The product was filtered, washed with deionized water, air-dried, and calcined in air at 300 °C for 2.5 h and 650 °C for 3.5 h. Finally, the white powder product was obtained and named Si-MCM-41.

#### Preparation of Ti-MCM-41

The tetrabutyl titanate was dissolved in the ethanol as the precursor of Ti. The mixture was stirred for 15–20 min. Then the MCM-41 was added into the solution. The MCM-41 was fully impregnated. Then the mixture was air-dried and calcined in air at 120 °C for 4 h and 550 °C for 5 h. Thus, the white powder product of mesoporous silica material was obtained and named Ti-MCM-41. The samples with different titanium loadings were obtained by changing the mole percentages of tetrabutyl titanate and MCM-41.

#### Preparation of Ti-V-MCM-41

A certain amount of ammonium metavanadate was firstly dissolved in dilute ammonia water. Then the Ti-MCM-41 was added into the solution and fully impregnated. Water and ammonia were evaporated. The obtained mixture was vacuum-desiccated at 100 °C for 3 h, and then calcined in air at 500 °C for 3 h. The samples with different vanadium contents were obtained by changing the mole percentages of ammonium metavanadate, tetrabutyl titanate, and MCM-41.

### Characterization

X-ray diffraction (XRD) patterns of the samples were obtained by using a Rigaku D/max-2500 X-ray diffractometer under Cu-K_α_ radiation at 40 kV and 100 mA. The diffraction data were collected every 0.02° at a scan speed of 1°(2*θ*)/min from 1° to 10° and 10° to 60°, respectively.

N_2_ adsorption–desorption isotherms were recorded at 77 K on a Micromeritics ASAP 2020. The samples were dried at 200 °C for 8 h before the measurement. BET surface areas were calculated from the linear part of the BET plot. The pores size distribution is calculated from desorption branche of the nitrogen adsorption.

Fourier-transform infrared spectroscopy (FT-IR) analysis were performed on a VERTEX 70 infrared spectrometer with KBr pellets in the infrared region of 4000 ~ 500 cm^−l^.

X-ray photoelectron spectroscopy (XPS) was measured on a VG ESCALAB5 multi-function electronic energy spectrometer with Al-K_α_ ray under CAE mode. The spectrometer was operated with a tube voltage of 9 kV and a tube current of 18.5 mA.

Transmission electron microscopy (TEM) experiments were conducted on a JEM-3010.

### Reaction procedure

The transesterification of DMC with phenol was conducted in a 100-mL three-neck round-bottomed flask under nitrogen atmosphere. In a typical experiment, a certain amount of phenol and catalyst were added into the flask under the stirring conditions at slowly increasing temperature. When the temperature reached 180 °C, DMC was introduced dropwise. The reaction temperature was maintained at 180 °C and the reaction mixture was treated under the refluxing condition at 180 °C. In the transesterification reaction, a distillate of DMC and methanol was collected slowly in a receiver flask attached to the liquid dividing head for analysis. After the reaction, the mixture was cooled to ambient temperature. The catalyst was regained by filtration and the filtrate was analyzed by gas chromatography.

### Product analysis

The azeotrope of DMC and methanol and the reaction system were analyzed by gas chromatography equipped with a capillary column (30 m) and a flame ionization detector (FID). The identification analysis of the reaction system was conducted on a 6890/5973 GC–Mass spectrometer.

## Results and discussion

### Catalyst structure

**Fig. 1:**
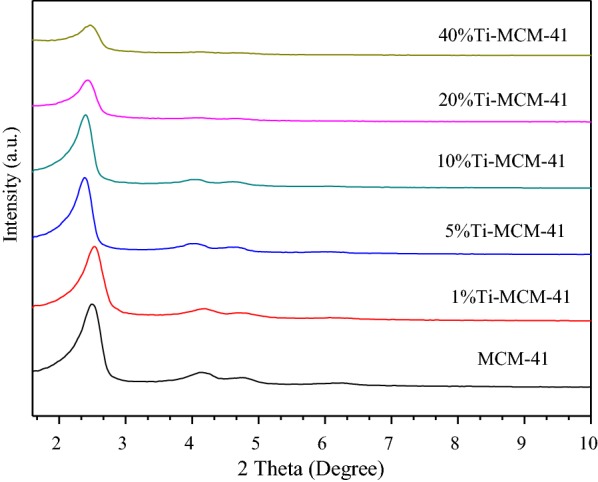
XRD patterns of the samples (small angle area)

**Fig. 2:**
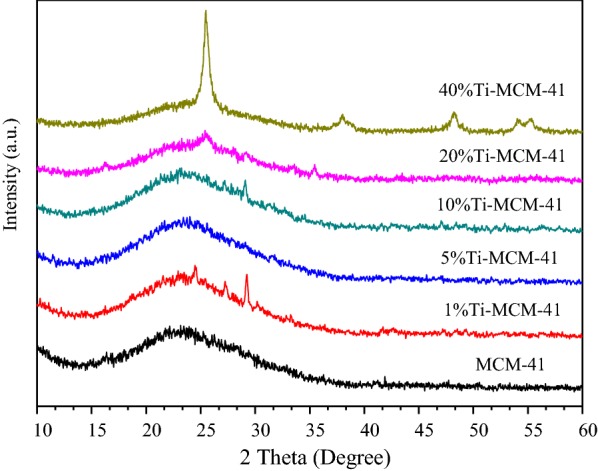
XRD patterns of the samples (big angle area)

The small-angle and big-angle XRD patterns are shown in Figs. 1 and 2, respectively. All the samples exhibit four well-defined peaks including a main reflection peak corresponding to the (100) plane at a low angle (around 2θ = 2–3°) and other three weak peaks which can be indexed with (110), (200), (210) planes at region of 2θ = 3–7°. It is similar to the MCM-41 standard spectrogram in the literature [14]. It can be seen from Fig. 1 that when the loading amount of titanium reaches 5%, the characteristic peak is generally shifted to the left. This is because titanium replaces silicon. According to the Bragg equation, the Ti–O bond grows longer than the Si–O bond, so the crystal surface spacing becomes larger, which indirectly indicates that the load of titanium enters the molecular sieve skeleton.

The strong and broad peak at *2θ* = 23° is observed in Fig. 2. The XRD pattern of Ti-MCM-41 is similar to that of MCM-41, indicating that the ordered hexagonal porous framework is retained after grafting titanium atom onto MCM-41. The slight changes of absorption peak intensity and peak position indicate that the metallic elements have entered into the framework of zeolite and changed the long-range order of silicon molecular sieve. In the XRD patterns of Ti-MCM-41, the characteristic peaks slightly decrease after loading titanium atom onto MCM-41. In contrast to Fig. 2, the spectra is not obviously changed even when the initial titanium loading increases up to 40 wt% in the synthesis solution, indicating that the titanium has been evenly dispersed into the catalyst.

**Fig. 3:**
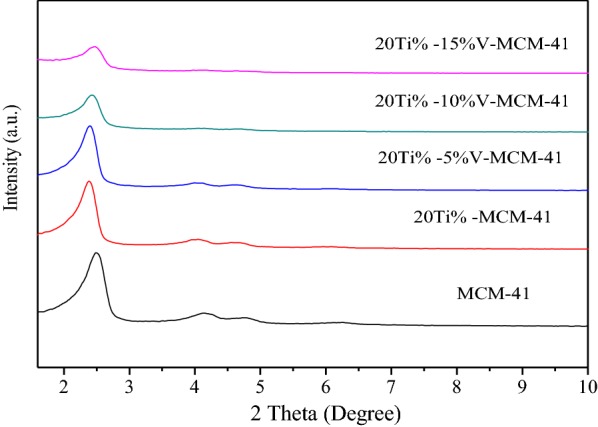
XRD patterns of the samples (small angle area)

**Fig. 4:**
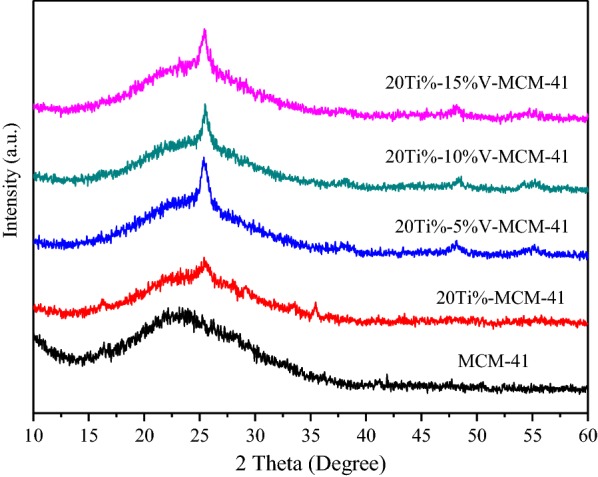
XRD patterns of the samples (big angle area)

The spectra of MCM-41 sample and catalyst samples Ti-V-MCM-41 containing active metals Ti and V were obtained. Figure 3 shows small-angle XRD spectra of the samples and Fig. 4 shows big-angle XRD spectra of the samples. The Ti and V contents have a great impact on the order of the crystal. With the increase of Ti and V content, not only the two weakly wide peaks (110) and (200) are gradually widened and even disappear, but the height of the main XRD peak (100) crystal surface is also gradually decreased, indicating that the incorporation of Ti and V are detrimental to the crystallization of the sample. Ti atomic radius and V atomic radius are larger than Si atomic radius, which makes the original long range of the all-silicon molecular sieve MCM-41 worse. When 20% Ti is loaded, the characteristic peak intensity of the samples is low. After the introduction of vanadium into Ti-MCM-41, the characteristic peak intensity of samples obviously reduces, indicating that introduced vanadium has the greater influence on the structure of samples. Vanadium changes the long-range order of silicon molecular sieve. As shown in the big-angle XRD spectra, after loading 20% Ti, a small peak appears. With the increase of vanadium, the small peak is increasingly obvious, thus changing the existence state of Ti. The characteristic peak of crystalline TiO_2_ can be seen obviously.

**Fig. 5:**
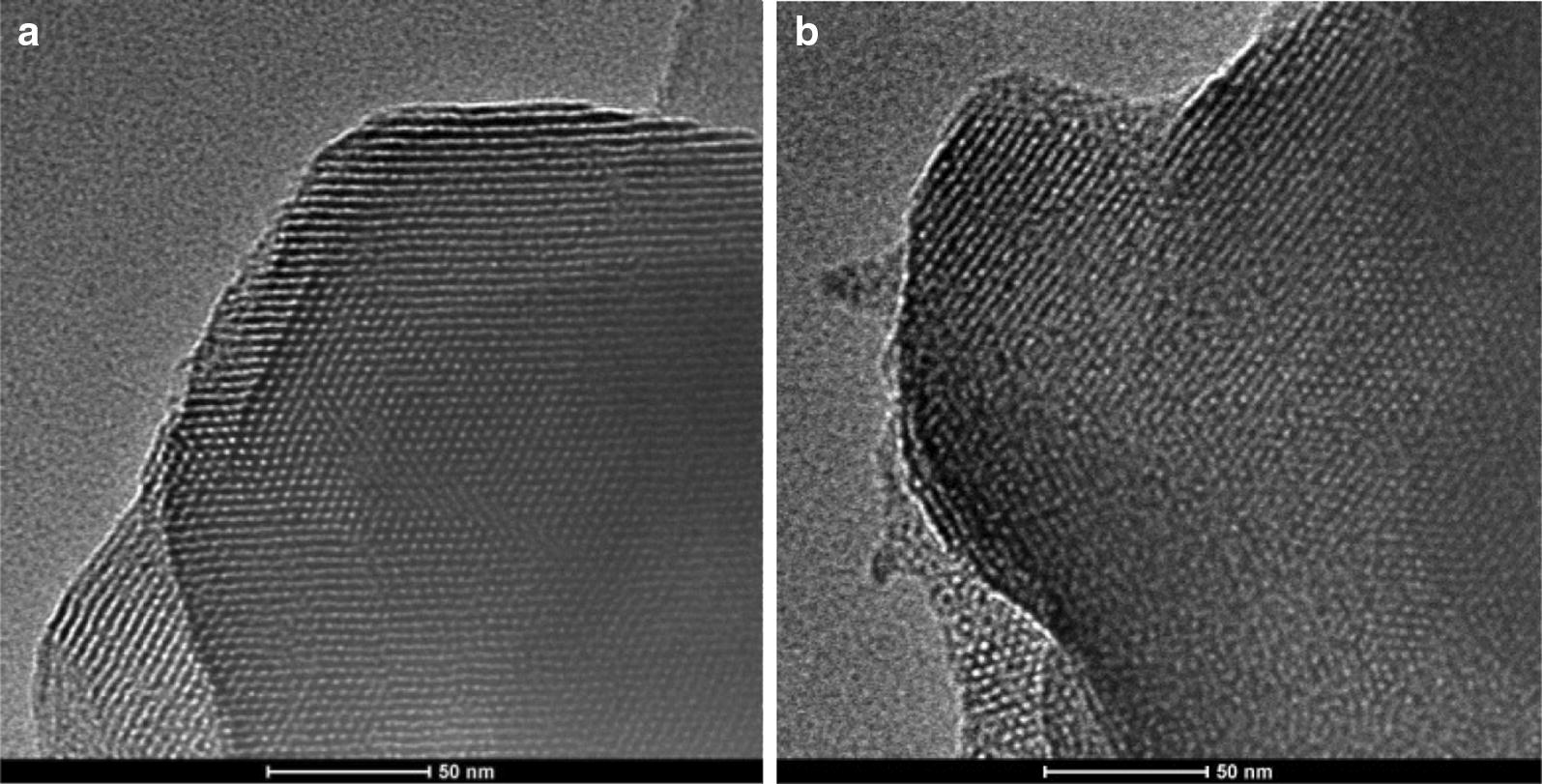
TEM images of MCM-41 (**a**), TEM images of 20%Ti-5%V-MCM-41 (**b**)

The morphology of the catalysts was observed by TEM, and the images are shown in Fig. 5. The TEM images clearly reveal that the microstructure of the samples was not changed after loading the active components of titanium and the original pore structure was maintained. The samples maintained relatively regular mesoporous structure, and these channels were stacked in parallel. With the introduction of titanium and vanadium, the regularity of mesoporous structure was reduced, and the parallel accumulation of pore channels in the TEM image was gradually unclear, which was consistent with the result of low-angle XRD.

### Infrared spectroscopy

**Fig. 6:**
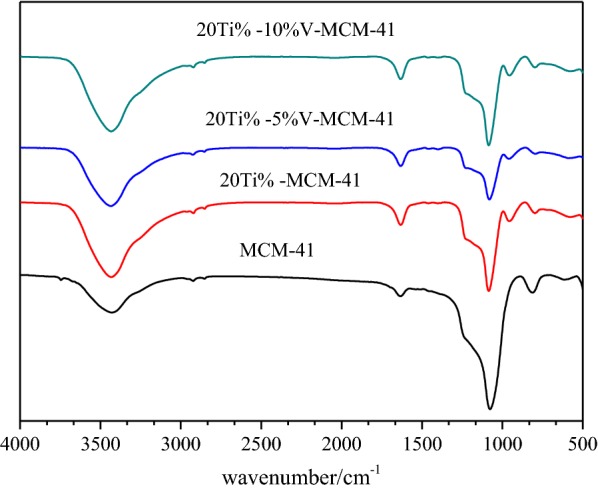
FT-IR spectra of the samples

FT-IR spectra are measured with KBr pellets in the infrared region of 400–4000 cm^−1^ (Fig. 6). The FT-IR spectra of all samples show the characteristic peaks of MCM-41. The feature peaks at about 1085 cm^−1^ are assigned to the asymmetric stretching vibration of Si–O–Si bond and the absorption peaks at about 800 cm^−l^ are caused by the symmetric stretching vibration of Si–O–Si bond. The asymmetric structure of molecular sieve is enhanced after the introduction of titanium and vanadium atoms into MCM-41. Simultaneously, distortion and vibration of the silicon oxygen tetrahedron are induced. In addition, vibration absorption peak corresponding to crystalline TiO_2_ is not found at 652 cm^−1^, indicating that titanium atom exists in the skeleton state. With the increase in Ti and V loadings, the sites of Si or O are occupied by Ti and V, thus leading to the gradually deceasing peak intensity at 1085 cm^−1^. All the data manifest that the mesoporous structure of MCM-41 is maintained without being destroyed after introducing titanium and vanadium atoms.

### Pore structure and specific surface area

The textural properties of the catalyst sample are listed in Table 1. All catalyst samples exhibited large specific surface areas, pore volumes, and pore sizes. The specific surface area, pore volume, and pore size of pure MCM-41 are respectively 1012 m^2^/g, 0.96 cm^3^/g, and 3.5 nm, whereas those of the loaded MCM-41 decrease slightly with the increase in titanium and vanadium atoms. However, the prepared Ti-MCM-41 and Ti-V-MCM-41 catalysts still have the larger specific surface areas, pore volumes and pore sizes. The decrease in the surface area, pore size and pore volume proved that titanium and vanadium atoms were introduced onto the internal surface of the mesoporous channels.

**Table 1:** Pore structure analysis of samples

Catalyst	S_BET_ (m^2^/g)	Pore volume (cm^3^/g)	BJH pore size (nm)
MCM-41	1012	0.96	3.5
20%Ti-MCM-41	965.43	0.91	3.32
20%Ti-5%V-MCM-41	923.32	0.85	3.12
20%Ti-10%V-MCM-41	879.61	0.79	2.86

### X-ray photoelectron spectroscopy analysis

**Fig. 7:**
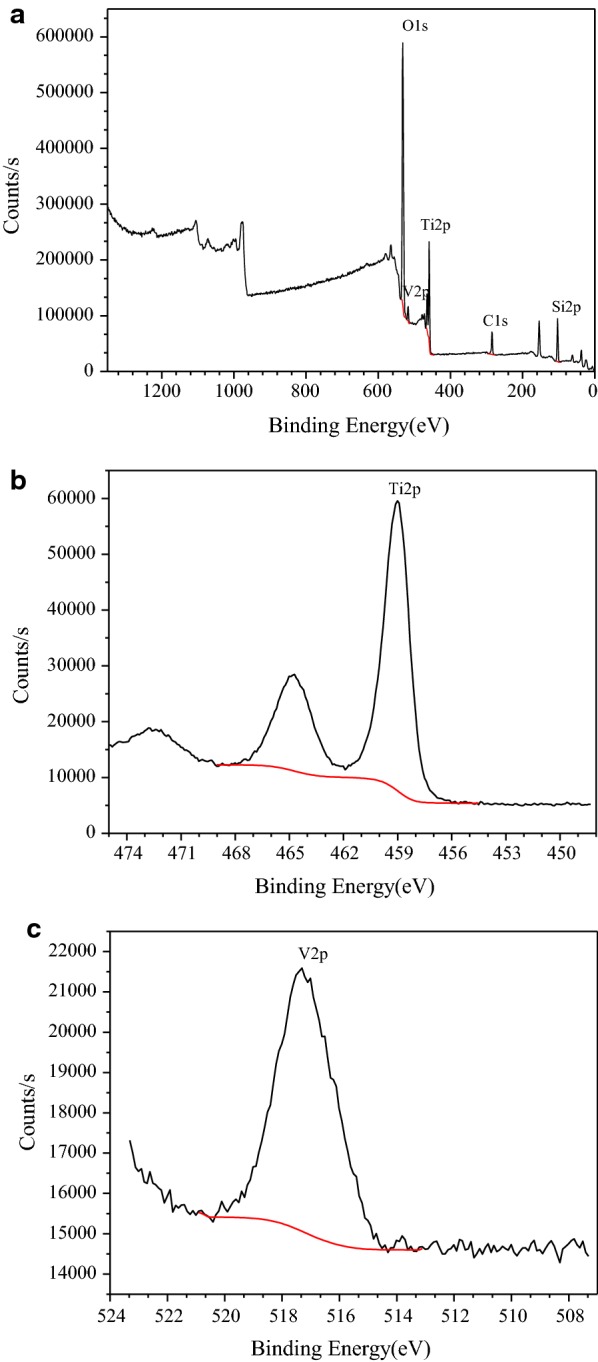
XPS spectra of 20%Ti-5%V-MCM-41 for the catalyst (**a**); XPS spectra of 20%Ti-5%V-MCM-41 for the catalyst (**b**); XPS spectra of 20%Ti-5%V-MCM-41 for the catalyst (**c**)

In order to obtain the chemical state information of the active components in the catalysts, XPS analysis was performed (Fig. 7). The XPS spectra of 20%Ti-5%V-MCM-41 (Fig. 7a) indicate the existence of the spectroscopic signatures of oxygen, vanadium, titanium, carbon and silicon. The Ti 2p3*/*2 binding energies of the TiO_2_/SiO_2_ catalysts were invariant with Ti loading in the range of 458.3 ± 0.2 eV, which was corresponded to the binding energy of titanium dioxide [16]. The two sharp peaks at 459.01 eV and 464.71 eV in Fig. 7b are respectively attributed to Ti 2P_3/2_ and Ti 2P_1/2_. Simultaneously, a strong peak of V 2P_3/2_ is observed at 517.31 eV (Fig. 7c). Taking the known binding energy of binary vanadium oxides into consideration, it was consistent with the material’s formal valence of + 5 [17]. The state of titanium is tetravalent titanium dioxide and the state of vanadium is vanadium pentoxide, as indicated by the standard spectra. The results indicated that titanium and vanadium atoms entered the molecular sieve framework. XPS results are well consistent with the above infrared spectrum analysis.

### Catalytic activity

The evaluation experiment of the catalyst was carried out in a reactive distillation unit. Reaction conditions were set as follows: phenol (30.0 g); DMC (30.0 g), catalyst (0.5 g), reaction temperature (180 °C), and reaction time (8 h). The conversion rate of raw materials is low in the blank experiment, whereas the conversion and selectivity are improved obviously after adding the catalysts.

**Fig. 8:**
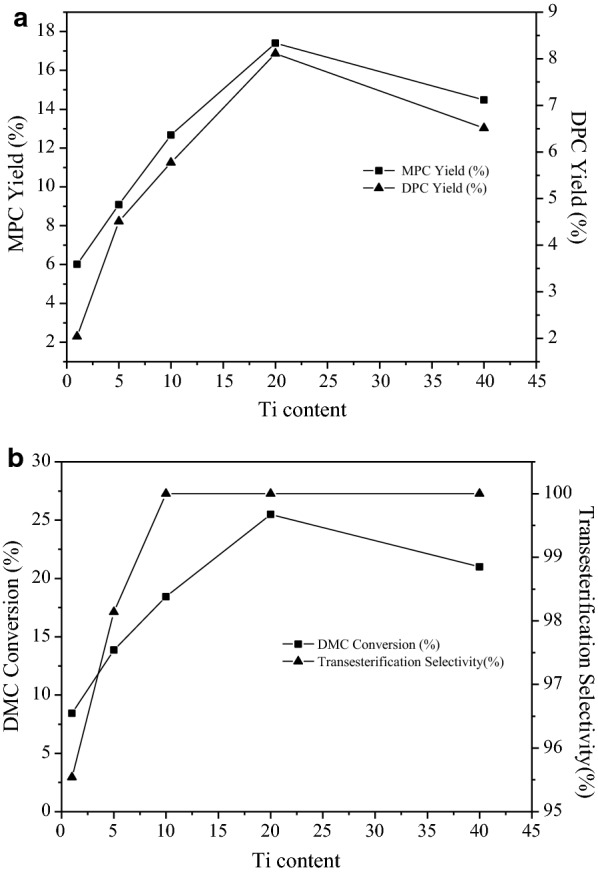
**a** Influence of Ti content on the catalytic activity. **b** Influence of Ti content on the catalytic activity

As shown in Fig. 8, after the introduction of active component titanium, the conversion rate of DMC, the yield of DPC and the yield of MPC firstly increase, and then decrease, displaying the same trend. The yield of MPC is greater than the yield of DPC. The selectivity of the catalyst for ester exchange reaction is good. The prepared catalysts have the better catalytic activity. With the increase in titanium content, the conversion and selectivity are increased. When the titanium content is 20%, the conversion and the selectivity is the best. When the titanium content increases to 40%, the conversion decreases. The analysis results of catalyst characterization also indicated that the formed partial TiO_2_ grains decreased the activity of the catalyst. The change also explained the relationship between the activity of catalyst and the structure and state of active metals. Too many TiO_2_ might block the carrier channel, reduce the specific surface area, and lower the activity. Therefore, the appropriate titanium content of catalyst should be selected so that the obtained catalysts have the perfect structure and good catalytic activity.

**Fig. 9:**
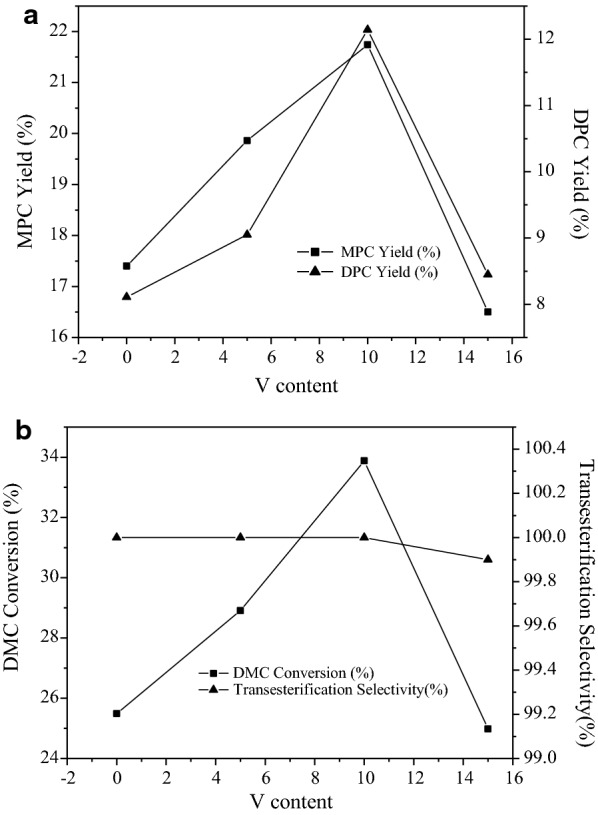
**a** The influence of V content on the catalytic activity; **b** the influence of V content on the catalytic activity

The catalyst containing 20% titanium was selected and then vanadium was loaded to prepare Ti-V-MCM41. The catalytic activity of the samples was evaluated (Fig. 9). With the introduction of active component vanadium, the conversion rate of DMC, the yield of DPC and the yield of MPC increased firstly and then decreased, displaying the same trend. The yield of MPC was greater than the yield of DPC. After the vanadium were loaded, the conversion and selectivity of samples were still good. With the increase in the content of vanadium, the conversion increased at first and then decreased slightly. The active component titanium and vanadium were loaded in MCM-41, obviously increased the catalytic activity and promoted the ester exchange reaction, displaying the good selectivity. However, too many V_2_O_5_ might block the carrier channel, reduce the specific surface area, and lower the activity. The characterization results of the catalysts indicated that the introduction of vanadium changed the existence state of partial titanium. An appropriate proportion of titanium to vanadium in the carrier allowed the best catalytic activity. The vanadium played a catalytic or coordinating role.

## Conclusion

In summary, a series of Ti-V-MCM-41 catalysts were prepared by grafting titanium and vanadium atoms on the surface of MCM-41. Ti-V-MCM-41 catalyst showed the excellent catalytic activity and selectivity for the transesterification reaction between phenol and DMC. Among the obtained catalysts, an appropriate amount of Ti (20%) and V (10%) supported on MCM-41 gave the best results to produce MPC and DPC in the yields of 21.74% and 12.14%. The mesoporous structure of MCM-41 molecular sieve was maintained in all samples and promoted the reaction selectivity. The different measurements indicated that the metallic elements (Ti) entered the framework of zeolite and formed Si–O–Ti bond. The titanium of catalyst still existed in the form of Ti(IV) species until Ti loading reached 20%. After the vanadium was introduced into Ti-MCM-41, it promoted the catalytic activity, reduced the by-products, and significantly improved the catalytic activity and stability of the catalysts. The vanadium pentoxide in the catalyst played a catalytic or coordinating role. In a word, the 10%V-20%Ti-MCM-41 catalyst is a promising catalyst for the transesterification of DMC and phenol.
